# Contribution of smoking and air pollution exposure in urban areas to social differences in respiratory health

**DOI:** 10.1186/1471-2458-8-179

**Published:** 2008-05-27

**Authors:** Tamara Schikowski, Dorothee Sugiri, Verena Reimann, Beate Pesch, Ulrich Ranft, Ursula Krämer

**Affiliations:** 1 Institut für Umweltmedizinische Forschung (IUF) at the Heinrich-Heine-University of Düsseldorf, Auf'm Hennekamp 50, 40225 Düsseldorf, Germany; 2 Berufsgenossenschaftliches Forschungsinstitut für Arbeitsmedizin (BGFA), Institute of Ruhr University Bochum, Buerkle-de-la-Camp Platz 1, 44789 Bochum, Germany

## Abstract

**Background:**

Socio-economic status, smoking, and exposure to increased levels of environmental air pollution are associated with adverse effects on respiratory health. We assessed the contribution of occupational exposures, smoking and outdoor air pollution as competing factors for the association between socio-economic status and respiratory health indicators in a cohort of women from the Ruhr area aged 55 at the time of investigation between 1985 and 1990.

**Methods:**

Data of 1251 women with spirometry and complete questionnaire information about respiratory diseases, smoking and potential confounders were used in the analyses. Exposure to large-scale air pollution was assessed with data from monitoring stations. Exposure to small-scale air pollution was assessed as traffic-related exposure by distance to the nearest major road. Socio-economic status was defined by educational level. Multiple regression models were used to estimate the contribution of occupational exposures, smoking and outdoor air pollution to social differences in respiratory health.

**Results:**

Women with less than 10 years of school education in comparison to more than 10 years of school education were more often occupationally exposed (16.4% vs. 10.1%), smoked more often (20.3% vs. 13.9%), and lived more often close to major roads (26.0% vs. 22.9%). Long-term exposure to increased levels of PM_10 _was significantly associated with lower school education. Women with low school education were more likely to suffer from respiratory symptoms and had reduced lung function. In the multivariate analysis the associations between education and respiratory health attenuated after adjusting for occupational exposure, smoking and outdoor air pollution. The crude odds ratio for the association between the lung function indicator FEV_1 _less than 80% of predicted value and educational level (<10 years vs. >10 years of school education) was 1.83 (95% CI: 1.22–2.74). This changed to 1.56 (95% CI: 1.03–2.37) after adjusting for occupational exposure, smoking and outdoor air pollution.

**Conclusion:**

We found an association between socio-economic status and respiratory health. This can partly be explained by living conditions indicated by occupational exposure, smoking behaviour and ambient air pollution. A relevant part of the social differences in respiratory health, however, remained unexplained.

## Background

Socio-economic status (SES) and exposure to increased levels of environmental air pollution are key issues in the discussion about social differences and health. Both have been linked to adverse health effects in recent years [1-3]. Socio-economic status is a determinant of health and is well known to play an important role in the development of several diseases, respiratory diseases among them [4-6]. There have been a number of studies regarding SES that have focused on respiratory symptoms and lung function decline [4,7,8]. At the same time, long-term exposure to air pollution has also been connected with a broad range of health effects, including respiratory impairments and lung diseases [9-12]. Because SES is considered as one of many important determinants of health, it has been included as a potential confounder or effect modifier in most epidemiological studies about adverse health effects of increased levels of air pollution [3,13,14]. Some recent studies have shown support for effect modification, with SES and air pollution-associated respiratory death [2,15,16].

In addition to environmental air pollution, possible SES-related risk factors for respiratory impairment are smoking, occupational exposure, malnutrition, low birth weight or multiple lung infections. These risk factors can be strongly influenced by SES: the level of education influences the type of occupation and income, which in turn influences the home living conditions [6]. The consideration of socio-economic factors is currently gaining greater interest in studies on environmental health, as social differences in environmental exposure may help to partly explain the observed differences in health [17]. SES may also modify the effect of environmental exposures by changing the susceptibility characteristics [18,19].

The aim of our analysis was to investigate the association between SES and respiratory impairment and whether or not this association could be ascribed to smoking or air pollution exposure that are associated with SES levels themselves. The present analysis was performed in order to determine the contribution of smoking and air pollution as explanatory factors for the relationship between SES and the development of respiratory symptoms. SES was taken as a function of the educational level, as education is the main determinant for occupational development and income in Germany. According to our knowledge this is the first study that analyses data of a German cohort of women with a focus on environmental differences and respiratory health.

## Methods

### Study population

We used data of a large cohort with detailed information regarding respiratory health, SES, and other risk factors. Details of that cohort study have been described previously [9,20]. Briefly, the baseline investigation of the SALIA (Study on the influence of Air pollution on Lung function, Inflammation and Aging) study group has been carried out between 1985 and 1994. These cross-sectional studies were part of the Environmental Health Surveys of the government of North-Rhine Westphalia, Germany, to obtain information regarding the effect of air pollution on respiratory health and allergies in women from the highly industrialized Ruhr district and two rural reference towns. These areas were chosen to represent a variety of environmental and socio-economic conditions. All women from predefined areas were asked to participate in the study. The study population comprised 4874 women aged 54–55 years at the time of baseline investigation and of German nationality. All participants gave informed consent for the interview, the health examination and the blood analysis. The response rate was stable throughout the consecutive cross-sections and was between 66% and 74%. Due to capacity reasons, only a subgroup, was invited to have their lung function measured (n = 2593). To avoid heterogeneity caused by specific social differences between rural and urban areas, only women from areas in the Ruhr district were included in the analysis (n = 3072). Additionally, we used only data from women with successful spirometry and with complete questionnaire data about education and covariates in the current analysis (n = 1251). The reason for this exclusion criterion was to be able to compare effects on lung function with effects on respiratory symptoms in the same study group.

The SALIA cohort study complies with the Helsinki Declaration and has received approval of the Ethics Committee of the Ruhr University in Bochum/Germany.

### Lung function measurements and COPD

Lung function was assessed by spirometry using VICATEST (VICATEST 4, Mijnhardt, Odijk, The Netherlands) by trained technicians. Identical protocols were used for all lung function measurements. The measuring instruments were calibrated prior to each session. At least two acceptable spirometric measurements were obtained from a minimum of four forced expirations. This procedure is in accordance with the ATS criteria [21]. In our analysis, we used the forced expiratory volume in one-second (FEV_1_) and the forced vital capacity (FVC). Linear regression models were used to predict the lung function parameter FEV_1 _and FVC based on age, height, race and sex. We used the equations which are recommended by the American Thoracic Society [22]. The prediction equations for creating reference values for these women were:

FEV_1_^predicted ^= 0.433-0.0036*age-0.00019*age^2^+0.000115*height^2^

FVC^predicted ^= -0.356 + 0.0187*age*0.00038*age^2 ^*0.000148*height^2^

We considered lung function to be impaired if FEV_1 _< 80% or FVC < 80% of the predicted value of the respective parameter. These cut-offs were also applied in the re-analysis of the Harvard Six City Study [13]. Furthermore, we applied the GOLD criterion [23] to identify chronic obstructive pulmonary disease (COPD). According to the GOLD criterion, a post-bronchodilator measurement of FEV_1_/FVC <0.7 defines COPD, at least stage one.

However, a post-bronchodilator measurement was not used in our study. Therefore, we excluded 44 women with bronchial asthma from further analysis of the association between COPD and SES and respiratory health risk factors, to avoid confounding of COPD with bronchial asthma. Bronchial asthma was considered present when diagnosed by a physician or if asthma medication was used.

### Respiratory symptoms and diagnoses, SES, and respiratory health risk factors

Symptoms and diagnoses were assessed by a self-administered standardised questionnaire. For the respiratory health status, the questionnaire included questions regarding chronic bronchitis and bronchial asthma diagnosed by a physician, frequent cough, and frequent cough with phlegm production as well as medication. Furthermore, relevant potential respiratory health risk factors such as occupational exposure to dust, gases, vapours, wet conditions or extreme temperatures, smoking, environmental tobacco smoke (ETS) and level of education were included in the questionnaire. All returned questionnaires were checked by the investigating physician. We grouped the women into three categories according to their reported current smoking habits: non-smoker (including ex-smoker), passive smoker (ETS at home and/or work place), and current smoker. We determined the SES by categorizing the women into three levels of education using the highest school level completed by either the women or her husband as low (< 10 years), medium (= 10 years), high (> 10 years).

### Air pollution data

We used ambient air concentration of particulate matter of less then 10 μm dynamic diameters (PM_10_) and nitrogen dioxide (NO_2_) to assess environmental pollution for our analysis. Total suspended particles (TSP) and NO_2 _are routinely measured by the State Environmental Agency of North Rhine Westphalia. The concentrations of NO_2 _are measured half-hourly by means of chemiluminescence. TSP were gathered with a low-volume sampler (air flow: 1 m^3^/h) and continuously measured using beta-ray absorption. To be able to compare our results with other air pollution studies where PM_10_, measurements were used, we multiplied the TSP measurements with a conversion factor of 0.71. This conversion factor was calculated from seven monitoring sites in the Ruhr area, where parallel measurements of TSP and PM_10 _were performed between 1998 and 2004 [20]. Across the Ruhr district, concentrations of ambient air pollutants were measured at stationary monitoring sites representing urban background levels. The monitoring stations are approximately located on an 8 km grid in the women's residential areas. Individual ambient air pollution exposure of the women was assessed by the measurements of the monitoring station nearest to the women's home addresses and by using 5-year-mean concentrations before the baseline investigations. We used 8 mean values from 6 monitoring stations. Furthermore, we used geographic information system (GIS) (Arc GIS 9.0, ESRI Redlands, Cato, U.S.A.) to calculate the distance of a woman's residential address to the nearest major road with more than 10.000 cars/day. A distance of less than 100 metres to the nearest major road was considered to indicate an increased exposure to local traffic-related air pollution. The daily traffic counts were obtained from the State Environmental Agency of North Rhine Westphalia.

### Statistical Analysis

The statistical analysis was performed by using SAS^® ^statistical software package, version 9.1.3 (SAS Institute, Cary, NC). The statistical description of respiratory health indicators and risk factors were provided per level of SES. We used the Cochran-Mantel-Haenszel test and linear regression for binary and continuous variables, respectively, to test a linear association of health outcomes and risk factors with the levels of SES. In order to test for a linear trend, SES was coded as follows:

One if educational level was less than 10 years school education, 0.5 if equal to 10 years and 0 if more than 10 years school education. An undirected association was assessed by chi-square test. The bivariate unadjusted associations between respiratory health indicators and risk factors were assessed by logistic regression. A stepwise procedure of multiple logistic regressions with pre-specified steps was used to analyse alterations of the associations between respiratory health indicators and SES (model I) by consecutively additional inclusion of occupational exposure (model II), tobacco smoke (model III) and environmental air pollution exposure (model IV) into the regression models. The results of the regression analyses are presented as odds ratios with 95% confidence limits and their p-values. The alteration of the crude SES effect by additional allowance of other risk factors in the model was assessed by the percentage reduction in the 'excess odds', i.e. OR-1, due to adjustment for the other risk factors. This percentage change can be taken as a measure of explained SES effect by other risk factors. Odds ratios of the continuous risk factors PM_10 _and NO_2 _were calculated for an interquartile range increase of 7 μg/m^3 ^and 16 μg/m^3^, respectively. The odds ratios presented for the SES factor indicate the risk ratios of the lowest versus the highest SES level. Interaction terms were included into the regression models to evaluate whether the risk factors modified the effect of the SES. Outcome variables which showed no association (p > 0.05) with SES were not analyzed. Likewise, risk factors, which showed no univariate association with SES, were not included in the analysis of alterations of the associations between respiratory health indicators and SES. If the association between SES and respiratory health was not homogeneous for different strata of the risk factors, the analysis was restricted to the stratum, where the association between SES and respiratory health indicator was highest.

## Results

### Prevalence of respiratory health impairment and SES

The cohort has been previously described in detail [9,20]. In brief, lung function measurements and complete information were obtained from 1251 women in the urban areas of the Ruhr district. In particular, information was available on school education of either the women or their husbands. The average age of the participants was 55 years (mean: 54.5, SD 0.6). Table 1 shows the distribution of SES expressed as educational levels. The prevalence of women with impaired respiratory health and a reduction in lung function according to their educational level are also presented in Table 1. The results show the differences in prevalence of respiratory impairment in women with different school education. Respiratory symptoms were more frequently reported in women with less than 10 years of school education. However, chronic bronchitis by a physician's diagnosis was more common in women with 10 years of school education. The percentage of women with impaired lung function (FVC and FEV_1 _<80% of predicted) was higher in the group with less than 10 years of school education. A similar result can be seen for COPD. The prevalence of impaired respiratory health decreased with increased education. A linear trend was seen for all respiratory health indicators with the exception of chronic bronchitis by a physician's diagnosis. For chronic bronchitis, no significant association of any kind with the educational level could be assessed using chi-square test. We therefore excluded chronic bronchitis from the further analysis. The association between respiratory health indicators and SES was stronger for the indicators based on lung function measurements than for those based on questionnaire variables. The percentage of women with reduced lung-function who, however, did not report symptoms was lower in better educated women. This might be due to selective underreporting of these women. For instance, 81% of women with more than 10 years of education and reduced lung function (FVC < 80%) reported frequent cough in comparison to only 67% of women with less than 10 years of education.

**Table 1:** Distribution of SES (educational level) in the study group and the prevalence of women with impaired respiratory health by SES

	**SES: all grades**		**<10 years**		**= 10 years of school education**		**>10 years**		
					
	**N**	**%**	**N**	**%**	**N**	**%**	**N**	**%**	
					
**SES**	1251	100	318	25.4	637	50.9	296	23.7	
**Indicators of impaired respiratory health**									**p-value^1 ^of linear trend effect of SES**

**Frequent cough**	1247	26.3	316	30.1	636	26.7	295	21.4	0.0152
**Frequent cough with phlegm production**	1240	12.9	312	16.0	633	12.3	295	10.9	0.0561
**Chronic bronchitis diagnosed by a physician**	1245	11.2	317	10.1	635	12.9	293	8.5	0.5785
**Bronchial asthma**	1238	3.6	312	4.8	632	3.6	294	2.0	0.0683
**COPD**	1207	4.1	303	5.3	614	4.9	290	1.4	0.0193
**FEV_1 _<80% predicted**	1251	20.5	318	24.5	637	20.9	296	15.2	0.0045
**FVC <80% predicted**	1251	26.8	318	33.3	637	26.2	296	21.0	0.0005

^*! *^Cochran-Mantel-Haenszel test

Abbreviations:

FEV_1_: Forced expiratory volume in 1 second; FVC: Forced vital capacity; COPD: Chronic obstructive pulmonary disease

### Prevalence of respiratory health risk factors and SES

The distributions of respiratory health risk factors in the study group are presented in Table 2. Unfavourable occupational exposure to dust, gases, vapours, wet conditions or extreme temperatures concerned 14.2% of the study group. Current smoking on entry to the study was in 17.5%, 36.6% were never smokers but did report environmental tobacco smoke (ETS) exposure, 45.9% were never smokers without environmental tobacco smoke, and 9.6% were ex-smokers. The mean distance to the nearest road with more than 10.000 cars per day was 519 m. 22.1% of all women lived in a distance of less than 100 m from a road with more than 10.000 cars a day (major road). The mean values for exposure to PM_10 _and NO_2 _were 49.4 μg/m^3 ^and 49.2 μg/m^3^, respectively. Women with less than 10 years of school education had a higher prevalence of occupational exposure. Women with lower SES had a higher exposure to ETS and were more likely to be current smokers. Further, women with less than 10 years of school education lived closer to major roads than women with higher education, but traffic exposure was not linearly associated with SES. Long-term exposure to increased levels of PM_10 _was significantly associated with lower school education. Women with less than 10 years of school education were exposed to higher levels of PM_10 _compared to women with higher education. The prevalence of occupational exposure and current smoking status and the long-term average concentration of PM_10 _were significantly increased with decreased level of education. NO_2 _exposure did not exhibit any association with SES. We therefore excluded the risk factor NO_2 _exposure from further analysis.

**Table 2:** Respiratory health risk factors by SES (educational level)

**Respiratory health risk factors**	**SES: all grades**		**<10 years**		**= 10 years of school education**		**>10 years**		**P-value^1 ^of linear trend effect of SES**
					
	**N**	**%**	**N**	**%**	**N**	**%**	**N**	**%**	
**Occupational exposure^2^**	1251	14.2	318	16.4	637	15.1	296	10.1	0.0296
**Current smoker**		17.5		20.3		17.9		13.9	0.0368
**Non-smoker with ETS**	1243	36.6	315	38.4	632	37.3	296	33.1	0.1780
**Non-smoker without ETS**		45.9		41.3		44.8		53.0	0.0038
**Distance to major road with >10.000 cars/day 0–100 m**	1216	22.1	304	26.0	624	19.9	288	22.9	0.3490

	**N**	**AM**	**N**	**AM**	**N**	**AM**	**N**	**AM**	
		**SD**		**SD**		**SD**		**SD**	

**PM_10 _5-years mean**	1254	49.4	318	50.0	637	49.7	296	48.2	<0.0001
		4.6		4.3		4.4		5.2	
**NO_2 _5-years mean**	1254	49.2	318	49.1	637	49.3	296	49.4	0.3941
		4.3		4.6		4.1		4.2	

^1 ^p-value of the logistic regression

^2 ^Dust, gases, vapours, wet conditions or extreme temperature

Abbreviations:

NO_2: _Nitrogen dioxide; PM_10_: Particulate matter with aerodynamic diameter of ≤ 10 μm, calculated as PM_10 _= 0.71*TSP; TSP: Total suspended particles; AM: Arithmetic mean; SD: Standard deviation

### Associations between respiratory health risk factors and respiratory health indicators

We tested all associations of risk factors with health outcomes in separate bivariate models (Table 3). Current smoking exhibited a significant adverse effect with all respiratory health indicators, whereas ETS did not. Likewise, occupational exposure was a significant respiratory health risk factor, but not significant for bronchial asthma. A reduction in FVC and FEV_1 _(<80% predicted) was significantly associated with exposure of high levels of PM_10_. An elevated prevalence of COPD and reduced FEV_1 _(<80% predicted) with women exposed to high traffic was indicated. The parameter estimates were similar but slightly smaller compared to those in our previous publication [9], where we included women from urban and rural areas. As exposure of ETS was not a risk factor for respiratory impairment in this study group we excluded ETS from further statistical analysis.

**Table 3:** Bivariate associations of respiratory health risk factors with respiratory health indicators by logistic regression

		**Frequent cough** **N = 1212**	**Frequent cough with phlegm production** **N = 1205**	**Bronchial asthma** **N = 1203**	**COPD** **N = 1172**	**FEV_1 _<80** **N = 1216**	**FVC <80** **N = 1216**
**Occupational exposure^1^**	OR	2.01	1.94	1.59	1.94	1.86	1.69
	95%CI	(1.44–2.82)	(1.28–2.95)	(0.75–3.38)	(0.97–3.90)	(1.30–2.66)	(1.20–2.37)
	p-value	<0.001	0.002	0.224	0.062	0.001	0.002
**Current smoker**	OR	2.03	1.99	2.34	3.32	2.22	1.47
	95%CI	(1.48–2.78)	(1.34–2.94)	(1.22–4.49)	(1.81–6.12)	(1.59–3.09)	(1.07–2.03)
	p-value	<0.001	0.001	0.011	<0.001	<0.001	0.018
**Non-smoker with ETS^2^**	OR	1.05	1.06	0.76	1.15	1.07	0.99
	95%CI	(0.78–1.41)	(0.71–1.58)	(0.36–1.63)	(0.55–2.42)	(0.78–1.49)	(0.75–1.32)
	p-value	0.740	0.772	0.479	0.706	0.674	0.966
**PM_10 _5-year mean [7 μg/m^3^]**	OR	1.06	1.09	0.94	1.25	1.46	1.44
	95%CI	(0.87–1.28)	(0.85–1.41)	(0.60–1.46)	(0.79–1.99)	(1.17–1.82)	(1.18–1.76)
	p-value	0.565	0.489	0.771	0.340	0.001	<0.001
**Distance to major road (< = 100 m) with >10.000 cars/day**	OR	1.13	1.02	1.04	1.69	1.30	1.07
	95%CI	(0.83–1.53)	(0.68–1.53)	(0.51–2.13)	(0.90–3.18)	(0.94–1.79)	0.79–1.45
	p-value	0.431	0.929	0.920	0.101	0.118	0.678

^1 ^Dust, gases, vapours, wet conditions or extreme temperature

^2 ^300 current smokers were excluded from the analysis

Abbreviations:

OR: Odds ratio; 95%CI: 95% Confidence interval; NO_2: _Nitrogen dioxide; PM_10_: Particulate matter with aerodynamic diameter of ≤ 10 μm, calculated as PM_10 _= 0.71*TSP; TSP: Total suspended particles; FEV_1_: Forced expiratory volume in 1 second; FVC: Forced vital capacity; COPD: Chronic obstructive pulmonary disease

### Association between SES and respiratory health indicators and its alteration by respiratory health risk factors

Before assessing the contribution of respiratory health risk factors to the association between SES and respiratory health indicators, we checked whether the effect between educational level and respiratory health risk factors was homogeneous. For chronic cough with and without phlegm production a significant (p < 0.1) modification of the SES effect by the distance to a major road was observed. The association was stronger in better educated women and stronger for women living in a distance of more than 100 m to the nearest major road. Therefore for the symptoms of chronic cough with and without phlegm production, we only considered the stratum of low traffic exposure, i.e. women living in a distance of more than 100 m to the nearest major road, in the following step-wise regression analysis. Table 4 shows the results of the step-wise regression analysis of the association between respiratory health indicators and SES adjusted by additionally including other risk factors (occupational exposure, current smoking and air pollution exposure). These results indicate that a low level of education was associated with a reduction in lung function, i.e. an increased prevalence of FEV_1 _or FVC less than 80% of the predicted value, as well as an increased prevalence of COPD, chronic cough with and without phlegm production, and bronchial asthma. The associations remained stable when adjusting for occupational exposure. However, the association was attenuated after additionally adjusting for smoking. After further adjustment for the environmental risk factors exposure of PM_10 _and distance to major road an additional attenuation of the SES effect was observed for COPD, FEV_1 _< 80% predicted and FVC <80% predicted. The explanatory measure of SES effects by other risk factors, the percentage reduction of excess odds ratio, showed a range between 5% to 10% attributable risk for the self-reported respiratory health indicators and 21% to 33% change for the measured lung function indicators. The smallest change was observed for bronchial asthma with current smoking showing the best explanation of the SES effect.

**Table 4:** Association between SES (educational level) and respiratory health indicators and its alteration by respiratory health risk factors Stepwise logistic regression analysis

		**Frequent cough^1^** **N = 944**	**Frequent cough with phlegm production^1^** **N = 938**	**Bronchial asthma** **N = 1203**	**COPD FEV_1_/FVC <0.7** **N = 1172**	**FEV_1_** **<80% N = 1216**	**FVC <80%** **N = 1216**
**Model I SES (educational level) unadjusted, 3 categories as continuous variables**	OR	2.01	2.02	2.33	2.46	1.83	1.99
	95%CI	(1.31–3.10)	(1.15–3.56)	(0.96–5.65)	(1.04–5.78)	(1.22–2.74)	(1.38–2.88)
	p-value	0.001	0.015	0.061	0.040	0.003	<0.001
**Model II SES adjusted for occupational exposure^2^**	OR	1.95	1.97	2.28	2.40	1.78	1.95
	95%CI	(1.26–3.00)	(1.11–3.48)	(0.94–5.56)	(1.01–5.68)	(1.18–2.68)	(1.35–2.83)
	p-value	0.003	0.020	0.068	0.046	0.005	<0.001
	%-change	6%	5%	4%	4%	6%	4%
**Model III SES additionally adjusted for current smoking**	OR	1.91	1.95	2.18	2.26	1.71	1.92
	95%CI	(1.23–2.97)	(1.10–3.46)	(0.89–5.32)	(0.94–5.41)	(1.13–2.58)	(1.32–2.79)
	p-value	0.004	0.023	0.088	0.068	0.011	0.001
	%-change	10%	7%	11%	14%	14%	7%
**Model IV SES additionally adjusted for 5-year mean PM_10_**	OR	1.92	1.92	2.27	2.20	1.59	1.79
	95%CI	(1.23–2.99)	(1.07–3.41)	(0.92–5.59)	(0.91–5.32)	(1.05–2.42)	(1.23–2.61)
	P-value	0.004	0.028	0.074	0.080	0.029	0.002
	%-change	9%	10%	5%	18%	29%	20%
**Model V SES additionally adjusted for distance to major road with >10.000 cars/day = 100 m**	OR	not applicable	not applicable	2.28	2.12	1.56	1.78
	95%CI			(0.92–5.62)	(0.88–5.10)	(1.03–2.37)	(1.22–2.60)
	P-value			0.074	0.095	0.037	0.003
	%-change			4%	23%	33%	21%

^1 ^Only women living in a distance more than 100 m to the nearest major road with heavy traffic

^2 ^Dust, gases, vapours, wet conditions or extreme temperature

Abbreviations:

OR: Odds ratio for <10 years education versus >10 years education; 95%CI: 95% Confidence interval; PM_10_: Particulate matter with aerodynamic diameter of ≤ 10 μm, calculated as PM_10 _= 0.71*TSP; TSP: Total suspended particles; FEV_1_: Forced expiratory volume in 1 second; FVC: Forced vital capacity; COPD: Chronic obstructive pulmonary disease; %-change: Calculated as the percentage reduction in the 'excess odds' due to adjustment for that factor

## Discussion

Our study is the first to analyze data of a cohort of women with the focus on environmental differences and respiratory health in Germany by including measurements of large- and small-scale air pollution. The results showed that women of lower educational level had a higher prevalence of respiratory impairment including a reduced lung function. We could observe that a reduction in lung functions (FEV_1 _and FVC < 80% of predicted value) was significantly associated with long-term exposure to high levels of PM_10_. Adjusting for smoking and outdoor air pollution decreased the relevance of SES for acquiring chronic respiratory symptoms or lung function impairment. The investigated respiratory health risk factors taken together, explained a relative portion of the social differences in the prevalence of self-reported chronic respiratory health indicators by 4% to 10% and for the measured respiratory health outcomes by 21% to 33%. The weaker explanatory potency of respiratory health risk factors for self-reported symptoms might partly be explained by a SES-dependent underreporting behaviour. Indeed, if the lung function measurements were taken as gold standard, we observed an increasing underreporting with decreasing educational level. Our findings are probably not only true for the Ruhr area, but can also be transferred to regions of other industrialized regions, as they might show similar social structures and patterns of behaviour. Our analysis is a contribution to the German environmental inequalities discussion by investigating the associations between air pollution exposure, including PM_10 _in ambient air, heavy traffic, occupational exposure, smoking behaviour, SES indicated by educational level, and respiratory health indicators including lung function measurements.

We used level of education, as it is widely accepted as a valid measure of SES [24] in particular in women. It is considered a good indicator of SES in women, because most women of this generation were not formally employed and had less occupational exposure [4]. Besides, social class, level of education and occupation are closely correlated and only some of the respiratory diseases may be caused by occupational exposure, however, women generally do not hold jobs with high levels of exposure to dust, such as miners, or extreme temperatures that are the potential causes of respiratory diseases [25].

It has been recognized that both air pollution and SES are priority areas for public health interventions [26]. At the same time, evidence showed that air pollution is a serious health concern [11,12]. A small number of studies in Germany showed that people with lower SES lived closer to major roads with high traffic exposure and had more adverse housing conditions and higher concentrations of indoor air pollution [27-30].

In our study we could show that impaired respiratory health was associated with low levels of education. This association however, attenuated after adjusting for smoking and ambient air pollution. Similar results were found in studies that focused on effect modification by SES of air pollution-associated mortality [2,4,16,31]. We observed effect modification by SES only for reported symptoms and traffic exposure, which might be related to differences in reporting behaviour. Several European studies revealed that low SES was not necessarily related to high exposure of air pollution [31,32]. It is possible that in different regions the social-group distribution varies.

We have to consider the following limitations when interpreting these results. The cross-sectional studies were designed to investigate the effect of high levels of air pollution on respiratory health. However, a cross-sectional study cannot address longitudinal issues.

Data on school education, lifestyle and other individual living conditions of these women were collected to control for potential confounders. Information on other attributes such as neighbourhood and income was not available. Another important limitation is that information regarding SES in early life, this was not available, therefore, it is possible when considering the total SES effect, then adjusting for height, and using a reference equation for FEV_1 _and FVC, there might be "adjusting out" an important intermediary process in the form of lung growth.

Clustering might play a role, when assessing SES effects. However, the Ruhr area is a very homogeneous area with respect to SES. In this study, controlling for clustering by area did not change standard deviations.

The assessment of SES was restricted to the highest achieved educational level by either the woman or her husband. 33.5% of husbands had higher educational levels than their spouses. We repeated the regression analysis by using only the educational level of the women (data not shown), however, the relevance of the education on respiratory health was weaker and the attenuation was less pronounced by the covariates for exposure.

Information bias might also have influenced the results for the reported respiratory diseases such as chronic bronchitis and frequent cough. Women with lower education might differently respond to questions regarding their respiratory health than people with a higher education. It is possible that women from low social status visit the physician less frequently compare to women of higher social status, so underreporting might have taken place. These could be the reasons for the missing association between chronic bronchitis by physician's diagnose and social status.

Small inequalities that might exist in exposure are likely to be the results of long-term socioeconomic processes whereby property values become depressed in areas of higher pollution and poorer people live in areas of low rent.

However, only small differences were detected in air pollution exposures between the groups, therefore it was difficult for the air variables to explain differences in SES effects. The coverage of the monitoring stations is probably inadequate to capture the spatial variations especially in NO_2_. This usually requires a very dense network. This may have contributed to the homogeneous exposures in NO_2, _but we included distance to a major road to capture smaller spatial variations.

We put together non-smokers and ex-smoker in the reference groups for the effect of current smoking with the potential consequence of confounding. However, in a separate analysis with ex-smoking as an additional risk factor, we did not see any substantial alterations of the association between SES and respiratory health indicators.

However, there are also several other strengths to our study. Firstly, we were able to differentiate between large- and small-scale measures of air pollution by using long-term exposure measures (5-year means) from stationary monitoring stations and the distance of the residential address to the nearest major road. Secondly, we only included women, men were excluded from this highly industrialized area, to avoid bias due to occupational exposures from mining and steel industry. The majority of these women were not in the work force and had no occupational exposures. Thirdly, the Ruhr district was historically the base of the coal mining and steel industry and had very high levels of ambient air pollution until the recent past, when this study was conducted. The decline in these industries was proportional throughout the area and homogeneously distributed across the whole study region.

## Conclusion

These findings demonstrate that the situation of women in Germany with regards to environmental differences is similar to conditions in other industrialized regions. There was a significant association between the low SES and the impaired respiratory health, which was indicated by increased prevalence of respiratory symptoms and diagnoses as well as reduced lung function. The effect of SES on respiratory symptoms and impaired lung function could partly be explained by known unfavourable living conditions, as could smoking and ambient air pollution, in those women with less school education. A considerable part of the social differences, however, remained still unexplained. Our results add to the environmental inequity discussion that low social status represents an important risk factor for developing respiratory diseases and that these women are more prone to live in higher polluted areas. High priority should be placed on reducing these risk factors for populations in less advantaged areas to promote environmental equity. Further follow-up of the cohort, currently being conducted, will provide additional information on these long-term effects of exposure to air pollution and the association with socioeconomic status and adverse health.

## Competing interests

The authors declare that they have no competing interests.

## Authors' contributions

TS performed the epidemiological analysis, drafted and wrote the paper. DS was co-investigator of the repeated cross-sectional studies, performed Geographical Information System analysis and was responsible for the data management and statistical analysis. VR helped to draft the manuscript. BP participated in the design of the study and helped to draft the paper. UR was co-investigator of the repeated cross-sectional studies, advised on exposure assessment, and commended on the statistical analysis and helped to draft the manuscript. UK was main investigator of the repeated cross-sectional studies, advised on exposure assessment, and commented on the statistical analysis and helped to draft the manuscript. All authors read and approved the final manuscript.

## Pre-publication history

The pre-publication history for this paper can be accessed here:


